# Spectrophotometric, Periodontal and Subjective Evaluations on Five Different Products for Clear Aligners Cleansing: Randomised Clinical Trial

**DOI:** 10.1155/ijod/9974833

**Published:** 2026-07-01

**Authors:** Maria Francesca Sfondrini, Maria Gloria Nardi, Maurizio Pascadopoli, Giancarla Alberti, Andrea Scribante

**Affiliations:** ^1^ Unit of Orthodontics and Pediatric Dentistry, Section of Dentistry, Department of Clinical, Surgical, Diagnostic and Pediatric Sciences, University of Pavia, Pavia, 27100, Italy, unipv.eu; ^2^ Department of Chemistry, University of Pavia, Via Taramelli 12, Pavia, 27100, Italy, unipv.eu; ^3^ Unit of Dental Hygiene, Section of Dentistry, Department of Clinical, Surgical, Diagnostic and Pediatric Sciences, University of Pavia, Pavia, 27100, Italy, unipv.eu

**Keywords:** absorbance, cleansing products, clear aligners, hygiene, periodontal parameters

## Abstract

This study aimed to assess five cleansing products and determine the most effective one for cleansing Invisalign CA in terms of absorbance, impact on periodontal parameters and subjective evaluation. The cleansing products evaluated in this study were drinking water (San Benedetto SpA, Scorzè, Italy), dishwashing soap (Svelto, Unilever Italia Mkt Operations S.r.l.; Rome, IT), effervescent tablets (Polident Antibacterial Denture Cleaner, Haleon plc, Weybridge, UK), crystals (Invisalign Cleaning Crystals, Align Technology, Inc.; San Jose, CA, USA) and gel (Geldis gel, Kalipharm S.r.l., Alba, IT). 20 patients treated with CA were enrolled and cleansing products were randomly assigned. Each patient was required to use the assigned product for 2 weeks until the next aligner change. When the aligner was changed, a different product was randomly assigned. Participants completed a questionnaire following their initial utilization and again after 1 and 2 weeks to assess their personal opinions regarding the cleansing products. The absorbance of the CA was measured using a spectrophotometer after 2 weeks of use of each cleanser. Data underwent statistical analysis (significance for *p*  < 0.05). Soap and crystals showed significantly higher values of absorbance (*p*  < 0.05). Regarding the periodontal indices and subjective perception of the visual and organoleptic characteristics of the CA, no statistically significant differences were detected (*p*  > 0.05). Gel, tablets and water exhibited lower absorbance values; subjective evaluation showed that water and crystals seemed to be associated with worse odour and taste than the other products, while gel and water seemed to cause an increase in opacity.

**Trial Registration:** ClinicalTrials.gov identifier: NCT06278116

## 1. Introduction

The advantages of clear aligners (CAs), beyond their orthodontic effects, include improved oral hygiene and more favourable periodontal health outcomes [1]. Several studies have reported that patients treated with CAs exhibit lower plaque index (PI) and bleeding on probing (BoP) scores compared with those wearing fixed orthodontic appliances, as well as a reduced risk of developing white spot lesions (WSLs) [2]. Nevertheless, adequate oral hygiene practices and proper cleansing of CAs remain essential to prevent plaque accumulation. In fact, CA surfaces present irregularities that facilitate bacterial adhesion and biofilm formation [3]. Moreover, aligners undergo physical and chemical changes during use, including abrasion and microfracture phenomena, which further promote bacterial deposition over time [4].

Patients are generally instructed to remove their CAs before brushing or flossing and prior to eating or drinking anything other than water [4]. However, it has been reported that many patients fail to remove aligners when consuming beverages or smoking, thereby increasing the risk of CA pigmentation and aesthetic deterioration [5].

The literature describes numerous products used for cleaning and disinfecting CAs, such as cleansing tablets, commercially available mouthwashes, liquid soaps, vinegar, dishwashing liquids, salt, sodium bicarbonate, and plain water [6]. Numerous studies have investigated the effectiveness of these cleansing methods using different analytical techniques [7–12]. Levrini and colleagues conducted two studies [8, 10] evaluating plaque accumulation and bacterial growth on CA surfaces using scanning electron microscopy (SEM) and bioluminometry. Three cleansing methods were compared—running water, effervescent tablets with sodium carbonate and sulphate crystals, and toothbrushing with toothpaste—with the latter showing the lowest bacterial load, while water alone was associated with higher levels of contamination.

Similarly, Lombardo et al. [9] performed a comparative SEM analysis of nine different cleansing strategies, combining rinsing water or detergents with sonic or ultrasonic baths. Their findings confirmed that water alone was associated with less effective cleansing outcomes, whereas the most successful approach employed ultrasonic treatment in combination with a 0.3% solution of benzalkonium chloride, a cationic germicidal detergent.

Other studies have focused on the effects of cleansing products on the physical and optical properties of aligner materials. One investigation [11] evaluated seven cleansing methods, including Invisalign cleaning crystals, Polident, Listerine mouthwash (Johnson and Johnson, New Brunswick, NJ, USA), vinegar, sodium hypochlorite, hydrogen peroxide, and toothbrushing with distilled water. While no significant differences were observed in surface roughness or flexural modulus, cleansing crystals, tablets, and mouthwash were associated with a smaller reduction in light transmittance. Similarly, Wible et al. [12] compared several cleansing agents on polypropylene/ethylene copolymer retainer material and concluded that no single method could be recommended as superior. However, although the material composition was comparable, the specimens used were not aligners, which may limit the direct applicability of these findings.

Bernard et al. [7] conducted a colorimetric and spectrophotometric evaluation of two cleansing products—Invisalign cleaning crystals and a Cordless Sonic Cleaner combined with Retainer Brite tablets (Dentsply Sirona Inc., York, PA, USA)—on three different CA brands subjected to typical staining substances, including coffee, red wine, black tea, and cola. Their results showed no significant differences in stain removal efficacy between the products, although Invisalign aligners appeared to be more prone to pigmentation. This observation was consistent with a previous in vitro study reporting higher absorbance values for Invisalign aligners after ageing compared with other CA brands [13]. Overall, the existing literature suggests comparable performance among different cleansing products. According to a systematic review, both over‐the‐counter and chemical cleaners exhibit antibacterial activity, although no clear superiority of a specific product has been established [6]. Conversely, some clinical trials have indicated that certain detergents are more effective than others.

A systematic review further suggests that toothbrushing with toothpaste should be performed carefully and thoroughly, ensuring that all surfaces and edges are adequately cleaned. Additionally, a combination of chemical and mechanical cleansing methods has been recommended to enhance CA hygiene [14]. More recently, Cen et al. [15] evaluated a novel combination of tannic acid and cetylpyridinium chloride, reporting effective inhibition of biofilm formation and reduction of pigmentation without compromising material safety.

Despite these findings, to the best of our knowledge, no clinical studies have simultaneously investigated the optical properties of CAs following the use of different home cleansing products, their effects on periodontal parameters, and patients’ subjective perceptions. To date, absorbance has been evaluated only in vitro, with limited clinical evidence available [13]. Absorbance, defined as the ability of a substance to absorb radiation at a specific wavelength [16], represents an indicator of an aligner’s capacity to maintain its optical and aesthetic properties over time. As absorbance is the logarithmic inverse of transmittance, higher absorbance values correspond to lower material transparency [17].

Although previous in vitro studies have analysed the spectrophotometric characteristics of different CA cleansing products [18, 19], no investigation has integrated these assessments with periodontal indices and patient‐reported outcomes. Accordingly, this study aimed to evaluate the efficacy of five distinct cleansing products for Invisalign CAs through both objective and subjective assessments. Specifically, absorbance, periodontal parameters and patients’ perceptions of visual and organoleptic characteristics were analysed. The first null hypothesis assumed that there would be no statistically significant differences in absorbance between the evaluated products. The second null hypothesis was that the periodontal parameters would not differ significantly between products. The third null hypothesis assumed that subjective perception, as evaluated through the questionnaire, would not differ significantly.

## 2. Materials and Methods

### 2.1. Trial Design

This was a single‐centre, randomised clinical trial, approved by the Unit Internal Review Board (registration number: 2022‐0504) [20]. CONSORT guidelines were used for structuring the manuscript. The completed CONSORT checklist is available in the Supporting Information (Table S1).

### 2.2. Participants

The study was conducted at the Unit of Orthodontics and Pediatric Dentistry, Section of Dentistry, Department of Clinical, Surgical, Diagnostic and Pediatric Sciences, University of Pavia, Pavia, Italy, starting in March 2024 and ending in July 2024. Participants signed an informed consent form before enrollment, and both interventions and outcome evaluations were performed within the same unit. Eligibility required a minimum age of 14 years and ongoing orthodontic treatment with Invisalign CAs. Individuals who did not adhere to the cleaning instructions or failed to complete the questionnaires were excluded.

### 2.3. Interventions and Outcomes

Patients were enrolled after signing the informed consent form (parents signed the consent form for minors). Around 2 weeks before the start of orthodontic treatment with Invisalign CA (Align Technology, Inc.; San Jose, CA, USA), professional manual and mechanical removal of supragingival and subgingival bacterial plaque was performed on both arches. At baseline (T0), the first aligner was provided to the patient, who was required to wear it for at least 22 h per day, except during meals and oral hygiene procedures. Patients were instructed to clean the CA with one of the five cleansing products selected for the study; participants were randomly allocated to a treatment protocol and supplied with a silicone‐bristled toothbrush (Kalipharm S.r.l., Alba, Italy) for the cleansing procedures. The five cleansing products were: drinking water (San Benedetto SpA, Scorzè, Italy), dishwashing soap (Svelto, Unilever Italia Mkt Operations S.r.l.; Rome, IT), effervescent tablets (Polident Antibacterial Denture Cleaner, Haleon plc, Weybridge, UK), crystals (Invisalign Cleaning Crystals, Align Technology, Tempe, Arizona) and gel (Geldis gel, Kalipharm S.r.l., Alba, IT). Manufacturers’ details and their compositions are shown in Table 1. Patients were required to use their assigned product for 2 weeks, following the instructions provided, until they changed to the next aligner. When the aligner was changed, a different material was randomly assigned, and patients were given different instructions.

**Table 1:** Cleansing products and their chemical composition.

Cleansing product	Manufacturer	Chemical composition
Water	San Benedetto SpA, Scorzè, Italy	—
Soap	Svelto, Unilever Italia Mkt Operations S.r.l.; Roma, IT	<5%. Amphoteric surfactants, DMDM Hydantoin, 5%–15% Anionic surfactants, Linalool, Perfume, Methylchloroisothiazolinone, Limonene, Methylisothiazolinone. Other constituents: aloe extract
Tablets	Polident Antibacterial Denture Cleaner, Haleon plc; Weybridge, UK	Citric acid, sodium carbonate, sodium bicarbonate, sodium carbonate peroxide, potassium caroate, TAED, sodium benzoate, PEG‐180, PVP/VA copolymer, sodium lauryl sulfoacetate, subtilisin, aroma, CI 42090, CI 73015
Crystals	Cleaning Crystals Invisalign, Align Technology, Inc.; San Jose, CA, USA	Sodium dichloroisocyanurate, sodium lauryl sulphate, sodium tripolyphosphate, sodium carbonate, sodium sulphate
Gel	Geldis gel, Kalipharm S.*r*.l.; Alba, IT	Phenoxyethanol, ethylhexylglycerin, dehydroacetic acid, benzoic acid, ascorbic acid, mentha piperita oil, limonene, sodium laureth sulphate, cocamide DEA, sodium chloride, water

A randomised crossover design was adopted, whereby each participant tested all five cleansing products according to a randomly generated allocation sequence. Each product was used for a 2‐week period in accordance with the manufacturer’s instructions provided for the study, followed by a 2‐week washout phase during which aligners were cleaned using water only. The 2‐week duration for each intervention was chosen to approximate the typical wear period of an aligner in clinical practice and to provide adequate time for potential changes in aligner optical properties, periodontal indices, and subjective outcomes to become detectable. At the same time, this duration minimised the influence of long‐term confounding factors. The 2‐week washout period was implemented to minimise potential carryover effects from the previously tested product and to allow the stabilisation of aligner surface conditions, as well as the use of a new aligner, before each subsequent cleansing protocol. Aligners used during the washout periods were not included in the spectrophotometric or periodontal analyses. Table 2 shows the instructions provided for each of the five products. A 19‐item questionnaire was administered to assess patients’ subjective perceptions of the cleansing material, including the organoleptic and visual features of the aligners and the ease of use of the cleaning protocol, following the first use and at 1‐ and 2‐week intervals. Scores ranged from 0 (no change) to 10 (remarkable change). The complete questionnaire is shown in Table S2 of the Supporting Information. The relative results and statistics are shown in Tables S3–S21 in the Supporting Information. After 2 weeks (T1), the patients were visited again: the questionnaire was compiled and the aligner was collected. Additionally, a periodontal evaluation of the Ramfjiord teeth (right maxillary first molar, left maxillary central incisor, left maxillary first premolar, left mandibular first molar, right mandibular central incisor, and right mandibular first premolar) [21] was performed recording the following indices: PI [22], BoP [23], probing pocket depth (PPD) [24], gingival index (GI) [25], basic erosive wear examination (BEWE) [26] and Schiff air index—sensitivity test [26]. The patient was then given another questionnaire and the following aligner, and a different cleansing product and toothbrush were randomly assigned. The procedure was repeated at 2 (T2), 4 (T3), 6 (T4) and 8 weeks (T5), considering a 2 week washout period between each cleansing protocol, until all the cleansing products had been tested. All products were masked, as described in Table 2. Absorbance was measured using a JASCO V‐750 spectrophotometer (Jasco Corporation, Tokyo, Japan) for each aligner after the application of the cleansing products. The following wavelengths were assessed: 300, 350, 400, 450 and 500 nm and a total range 300–500 nm (λ _TOT_). A control value was obtained using a new, uncontaminated aligner. Reflectance measurements acquired via spectrophotometry were subsequently converted into absorbance values.

**Table 2:** Instructions for the use of the cleansing products.

Cleansing product	Instructions
Water	The product is supplied in an unlabelled spray bottle. Participants were instructed to apply it once daily for 14 days by spraying it onto the aligner, followed by cleaning with a soft‐bristled toothbrush for 1 min and subsequent rinsing under running water.
Soap	Supplied in a clear bottle without labelling, the product is used once per day over a 14‐day period. It is applied to the aligner, which is then brushed for 1 min using a soft‐bristled toothbrush and subsequently rinsed with running water.
Tablets	Blinding was achieved by covering all labels on the packaging with a black permanent marker. Over a 14‐day period, patients prepared a solution by dissolving a tablet in warm water (30–35°C), immersed the aligner for 5 min, and then rinsed it thoroughly under running water.
Crystals	To ensure masking, all labels on the packaging were marked with a black permanent marker. The cleansing procedure consisted of dissolving the crystals in a glass of warm water (30–35°C), stirring for 20 s, and then immersing the aligner for 15 min. After soaking, the aligner was rinsed under fresh running water. This protocol was repeated daily for 14 days.
Gel	Blinding was achieved by marking all labels on the packaging with a black permanent marker. The cleansing procedure consisted of applying the product onto the aligner with a soft toothbrush for 1 min, followed by rinsing under fresh running water. This protocol was repeated daily for 14 days.

All clinical periodontal parameters were assessed by a single calibrated examiner, while spectrophotometric measurements were performed by a trained operator using a standardised protocol. The questionnaire data were self‐reported by the participants at predefined time points.

No missing data were observed as all enrolled participants completed the study and provided complete datasets for all outcomes. Therefore, no imputation procedures were required.

Finally, no deviations from the approved study protocol occurred during the trial. All interventions, follow‐up visits, and outcome assessments were conducted as originally planned and registered.

### 2.4. Sample Size

Sample size estimation (*α* = 0.05 and power = 80%) was performed considering absorbance as the primary endpoint. Based on prior findings [13], a clinically meaningful difference of 0.0188 in CA absorbance among groups was assumed, with an expected mean of 0.190 and a standard deviation of 0.03 for the first group after 2 weeks. Given that each participant provided one CA for each cleansing protocol, 40 aligners per method were required, leading to a total of 160 aligners across the four washout phases.

### 2.5. Randomisation and Blinding

A block randomisation approach was employed for participant allocation, with the random sequence generated by the data analyst using permuted blocks of 20 individuals. Enrollment, delivery of standardised verbal instructions, and outcome recording were carried out by a staff member not involved in the earlier phases of the study. Allocation concealment was ensured through the use of pre‐prepared, sequentially numbered, opaque, sealed envelopes (SNOSE), which also concealed the identity of the product intended for home use. At each follow‐up visit, participants received different products following a 2‐week washout period between interventions. The data analyst remained blinded to both group allocation and study outcomes. To maintain blinding, all CA cleansing products were provided in identical or unlabelled containers, with the original labels removed or obscured. Consequently, neither the clinicians nor the participants were aware of the assigned treatments.

### 2.6. Statistical Methods

All statistical analyses were conducted with R software (version 3.1.3; R Foundation for Statistical Computing, Vienna, Austria). Summary statistics included mean, standard deviation, median and range (minimum–maximum). The normality assumption was verified using the Kolmogorov–Smirnov test. Repeated‐measures ANOVA was applied to assess differences across variables, with Tukey’s post hoc test used for pairwise comparisons. Statistical significance was defined as *p* < 0.05.

## 3. Results

### 3.1. Participants Flow and Baseline Data

**Figure 1:**
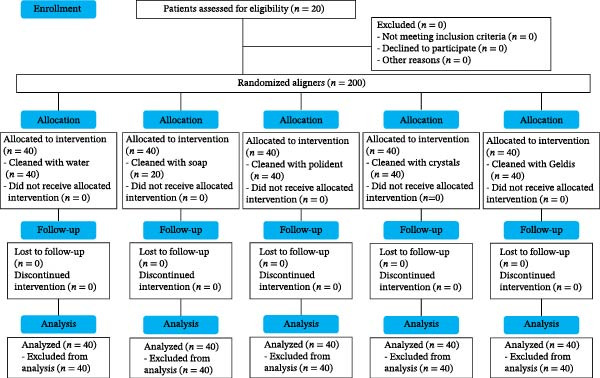
CONSORT flow diagram.

The study flow diagram is presented in Figure 1. Twenty participants were included, and no dropouts occurred. At baseline, the average age of the sample was 21.31 ± 6.20 years, comprising 14 female participants (mean age 22.90 ± 6.39) and six male participants (mean age 17.31 ± 3.16).

Descriptive and inferential statistics for the study variables were expressed using a letter‐based notation system [27].

### 3.2. Spectrophotometric Evaluation Through Absorbance (*A*)

Absorbance scores are set out in Table 3. Statistically significant higher absorbance values for the total wavelength analysed were found for soap and crystals in respect to the other products (*p* < 0.05). Absorbance values for the other wavelengths resulted not significant (*p* > 0.05). It is important to emphasise that better optical clarity, reflected by lower absorbance values, does not equate to superior microbiological decontamination. A cleanser may effectively remove or inhibit biofilm formation while still producing changes in material surface characteristics that influence light transmission and conversely. Optical measures alone cannot be used to infer the antimicrobial performance, which depends on biochemical interactions not captured by spectrophotometry. Consequently, the optical results of this study should be considered complementary rather than indicative of hygienic efficacy.

**Table 3:** Mean ± standard deviation (SD) values of absorbance at different wavelengths (nm).

Product	Wavelength (nm)											
	300 nm		350 nm		400 nm		450 nm		500 nm		λ_TOT_ nm	
	Mean	SD	Mean	SD	Mean	SD	Mean	SD	Mean	SD	Mean	SD
Water	1.44^a^	0.07	1.41^a^	0.08	1.46^a^	0.08	1.47^a^	0.08	1.46^a^	0.07	0.04^a^	0.02
Soap	1.43^a^	0.11	1.42^a^	0.12	1.50^a^	0.12	1.45^a^	0.11	1.45^a^	0.10	0.07^b^	0.06
Tablets	1.50^a^	0.19	1.47^a^	0.13	1.50^a^	0.11	1.50^a^	0.11	1.50^a^	0.11	0.04^a^	0.02
Crystals	1.49^a^	0.13	1.46^a^	0.14	1.48^a^	0.13	1.49^a^	0.13	1.48^a^	0.12	0.06^b^	0.04
Gel	1.46^a^	0.17	1.43^a^	0.14	1.47^a^	0.13	1.48^a^	0.13	1.48^a^	0.14	0.03^a^	0.01
Control	1.50^a^	0.19	1.47^a^	0.13	1.50^a^	0.11	1.50^a^	0.11	1.49^a^	0.11	0.03^a^	0.01

*Note*: The control group represents a new, unused aligner and was included as a reference for optical properties. Different superscript letters within the same column indicate statistically significant differences among cleansing products (ANOVA followed by Tukey’s post hoc test; *p* < 0.05).

Abbreviation: λ_TOT_, total absorbance (300–500 nm).

### 3.3. Periodontal Parameters

Regarding PPD, PI, GI, BoP, BEWE, and SAI (Table 4), no significant intergroup differences have been found (*p* > 0.05).

**Table 4:** Mean ± standard deviation (SD) values of periodontal parameters recorded at baseline and after the use of each cleansing product.

Product	Parameter											
	PPD (mm)		PI		GI		BoP		BEWE		SAI	
	Mean	SD	Mean	Mean	Mean	SD	Mean	SD	Mean	SD	Mean	SD
Baseline	1.73^a^	0.43	0.61^a^	0.74	0.22^a^	0.42	0.19^a^	0.39	0.00^a^	0.00	0.13^a^	0.44
Water	1.79^a^	0.49	0.48^a^	0.64	0.04^a^	0.19	0.20^a^	0.41	0.04^a^	0.19	0.11^a^	0.46
Soap	1.84^a^	0.63	0.33^a^	0.61	0.09^a^	0.29	0.20^a^	0.41	0.00^a^	0.00	0.11^a^	0.46
Tablets	1.57^a^	0.46	0.48^a^	0.64	0.02^a^	0.14	0.09^a^	0.29	0.00^a^	0.00	0.09^a^	0.35
Crystals	1.89^a^	0.59	0.59^a^	0.69	0.06^a^	0.30	0.26^a^	0.44	0.00^a^	0.00	0.07^a^	0.33
Gel	1.76^a^	0.44	0.52^a^	0.75	0.11^a^	0.32	0.15^a^	0.36	0.00^a^	0.00	0.09^a^	0.45

*Note*: Different superscript letters within the same column indicate statistically significant differences among cleansing products (ANOVA followed by Tukey’s post hoc test; *p* < 0.05).

Abbreviations: BEWE, basic erosive wear examination; BoP, bleeding on Probing; GI, gingival Index; PI, plaque Index; PPD, probing pocket depth; SAI, Schiff air index.

### 3.4. Questionnaire

Concerning the questions about the subjective appreciation of the cleansing products, no statistically significant differences were detected between groups (*p*  > 0.05). The complete results of the statistical comparisons between the 19 questions in the questionnaire are shown in Tables S3–S21 of the Supporting Information.

### 3.5. Harms and Adverse Effects

No adverse events or harmful effects were reported throughout the study.

## 4. Discussion

CA cleansing and disinfection are essential for maintaining adequate oral hygiene and periodontal health during orthodontic treatment [28]. Although patients undergoing fixed orthodontic therapy generally exhibit greater plaque accumulation and higher levels of cariogenic bacteria [29], the occurrence of WLSs has also been documented in patients treated with CAs [30]. In the absence of proper oral hygiene, CA therapy may therefore be associated with an increased risk of enamel demineralisation and WLSs, similar to those of other orthodontic modalities [31].

Several cleansing products have been developed to evaluate their effects on periodontal health as well as the visual and organoleptic characteristics of CAs. In the present study, five cleansing products—water, soap, tablets, crystals and gel—were compared. The findings led to a partial rejection of the first null hypothesis, given the presence of statistically significant differences in absorbance over the total wavelength range, with soap and crystals showing higher absorbance values than the other products (*p* < 0.05). Based on these findings, gel, tablets and water were associated with more favourable optical outcomes. However, the statistically significant differences for soap and crystals were limited to the total wavelength range and the 500 nm wavelength, making it uncertain whether these variations are sufficient to justify contraindicating their use. Changes in absorbance at isolated wavelengths do not necessarily correspond to clinically perceptible differences or reduced performance during daily aligner use.

An important consideration is whether the observed absorbance variations translate into visually perceptible colour or transparency changes. Although absorbance reflects modifications in the optical properties of aligner materials, clinically detectable aesthetic alterations occur only when human visual perception thresholds—commonly quantified as ΔE values within the CIELAB colour space—are exceeded [32]. Because absorbance values cannot be directly converted into ΔE values without complementary colorimetric analyses, the clinical perceptibility of the differences observed in this study remains uncertain. Consequently, statistically significant absorbance variations should be interpreted with caution as they may not correspond to clinically relevant aesthetic changes. Future studies integrating spectrophotometry with ΔE‐based colour evaluation would help clarify the perceptibility thresholds for aligner materials.

Considering the available literature, cleansing with running water alone appears suboptimal and should be discouraged [8–10]. Previous studies have employed a wide range of analytical techniques to assess CA cleansing protocols, including SEM [8, 9], bioluminometry [10], CIELAB‐based colorimetric analysis [7], and spectrophotometry [7, 13], as applied in the present study. The heterogeneity of methodologies, staining agents, and experimental conditions limits comparability and hinders the standardisation of recommendations. Future research should therefore integrate microbiological, optical and microscopic assessments to provide a more comprehensive evaluation of CA cleansing protocols.

The results confirmed the second null hypothesis as periodontal parameters did not differ significantly across the tested cleansing products.

To date, no clinical studies have specifically investigated periodontal outcomes in CA patients in relation to different cleansing methods, limiting direct comparisons. The lack of significant differences may be attributed to several factors, including professional plaque removal and standardised oral hygiene instructions provided at baseline, the short duration of each intervention phase (2 weeks), the young age of the participants, and their generally good periodontal health. These conditions likely reduced baseline variability and the sensitivity of periodontal indices to detect subtle or progressive changes. Therefore, the absence of intergroup differences should be interpreted in this context rather than as evidence of equivalent long‐term periodontal effects.

The third null hypothesis was supported since no statistically significant differences were found in patients’ subjective evaluation of the visual and organoleptic features of the CAs. The questionnaire assessed aligner smell, taste, surface roughness, colour changes, transparency, opacity, adherence, ease of use, cleansing time and frequency, and perceived changes in salivation. Although no statistically significant differences emerged, clinically relevant trends were observed. Soap, tablets and gel were generally perceived as more pleasant in terms of smell and taste, while drinking water was more frequently associated with increased surface roughness. Transparency was rated positively across all products, although gel and water were, on average, associated with increased opacity. All cleansing methods were considered easy to use, but tablets and crystals required longer cleansing times, and drinking water alone was associated with the need for more frequent daily cleansing. Given the absence of previous studies evaluating patient‐reported outcomes related to CA cleansing, comparisons with the existing literature are not currently feasible.

Several limitations should be acknowledged. Home cleansing procedures may have been influenced by patient compliance, and the aligner change interval adopted in this study differs from the current clinical practice, which often involves changes every 7–10 days. Moreover, different CA brands exhibit distinct optical and surface properties and varying susceptibility to pigmentation [13, 32–42]; thus, limiting the analysis to Invisalign aligners reduces the generalizability of the findings. Complete blinding could not be ensured as some products may have been recognisable by appearance, odour or mode of use, potentially introducing expectation bias in subjective assessments.

From a methodological perspective, the statistical analysis did not explicitly account for within‐subject correlations, period effects or interaction effects inherent to crossover designs. Despite the inclusion of a washout period and the availability of a complete and balanced dataset, more sophisticated approaches, including repeated‐measures ANOVA or mixed‐effects models, could provide a more reliable assessment in future research. Additional limitations include the absence of microbiological or SEM‐based validation of cleanliness, the subjectivity of the questionnaire data, and the relatively short duration of each intervention period, which may have limited the detection of subtle or long‐term effects.

Future research should adopt aligner wear intervals consistent with current clinical protocols, integrate microbiological and surface analyses, and include different CA brands to enhance the external validity of the findings.

## 5. Conclusions

According to the present study, significantly higher absorbance values were found for soap and crystals, while gel, water and tablets showed lower values. No significant effect was found on periodontal parameters. From subjective evaluation, drinking water and crystals seem to be associated with a worse odour and taste than the other products, while gel and drinking water seem to cause an increase in opacity. Therefore, evidence from both the existing literature and the present study indicates that tablets and gel are suitable options for CA cleansing.

## Author Contributions

Conceptualisation, methodology: Andrea Scribante and Maria Francesca Sfondrini. Software, formal analysis: Andrea Scribante. Investigation: Maria Gloria Nardi and Giancarla Alberti. Resources: Maria Francesca Sfondrini. Data curation: Maria Gloria Nardi and Maurizio Pascadopoli. Writing – original draft preparation: Maria Gloria Nardi and Maurizio Pascadopoli. Writing – review and editing, visualization, supervision, project administration: Andrea Scribante and Maria Francesca Sfondrini.

## Funding

No funding was received for this manuscript. Open access publishing facilitated by Universita di Pavia, as part of the Wiley ‐ CRUI‐CARE agreement.

## Disclosure

All authors have read and agreed to the published version of the manuscript.

## Ethics Statement

Written approval and informed consent were obtained.

## Conflicts of Interest

The authors declare no conflicts of interest.

## Supporting Information

Additional supporting information can be found online in the Supporting Information section.

## Supporting information


**Supporting Information** CONSORT 2025 checklist is shown in Table S1 of the Supporting Information. The complete questionnaire is shown in Table S2 of the Supporting Information. The relative results and statistics are shown in Tables S3–S21 of the Supporting Information.

## Data Availability

The data that support the findings are available within the manuscript.
