# Supplementary material for: Spectrophotometric, Periodontal and Subjective Evaluations on Five Different Products for Clear Aligners Cleansing: Randomised Clinical Trial
Source: Int J Dent. 2026 Jul 1;2026:9974833. doi: 10.1155/ijod/9974833 (PMC13323852; doi:10.1155/ijod/9974833)
Supplement: Supplementary file 1 — Supporting Information CONSORT 2025 checklist is shown in Table S1 of the Supporting Information. The complete questionnaire is shown in Table S2 of the Supporting Information. The relative results and statistics are shown in Tables S3–S21 of the Supporting Information. [file IJOD-2026-9974833-s001.zip › Supplementary Materials.docx]

**1. Questionnaire**

| Have you perceived a change in the smell of the aligner after cleaning?  (0=no change, 10=remarkable change) | | | | | | | | | |
| --- | --- | --- | --- | --- | --- | --- | --- | --- | --- |
| 1 | 2 | 3 | 4 | 5 | 6 | 7 | 8 | 9 | 10 |
| How do you rate the smell of the aligner after cleaning? (0=terrible, 10=very pleasant) | | | | | | | | | |
| 1 | 2 | 3 | 4 | 5 | 6 | 7 | 8 | 9 | 10 |
| How much has the smell of the aligner improved? (0=not at all, 10=very much) | | | | | | | | | |
| 1 | 2 | 3 | 4 | 5 | 6 | 7 | 8 | 9 | 10 |
| How much has the smell of the aligner worsened? (0=not at all, 10=very much) | | | | | | | | | |
| 1 | 2 | 3 | 4 | 5 | 6 | 7 | 8 | 9 | 10 |
| Have you perceived a change in the taste of the aligner after cleaning?  (0=no change, 10=remarkable change) | | | | | | | | | |
| 1 | 2 | 3 | 4 | 5 | 6 | 7 | 8 | 9 | 10 |
| How do you rate the taste of the aligner after cleaning? (0=terrible, 10=very pleasant) | | | | | | | | | |
| 1 | 2 | 3 | 4 | 5 | 6 | 7 | 8 | 9 | 10 |
| How much has the taste of the aligner improved? (0=not at all, 10=very much) | | | | | | | | | |
| 1 | 2 | 3 | 4 | 5 | 6 | 7 | 8 | 9 | 10 |
| How much has the taste of the aligner worsened? (0=not at all, 10=very much) | | | | | | | | | |
| 1 | 2 | 3 | 4 | 5 | 6 | 7 | 8 | 9 | 10 |
| Have you perceived a change in the roughness of the aligner after cleaning?  (0=no change, 10=remarkable change) | | | | | | | | | |
| 1 | 2 | 3 | 4 | 5 | 6 | 7 | 8 | 9 | 10 |
| How do you rate the roughness of the aligner after cleaning? (0=very rough, 10=very smooth) | | | | | | | | | |
| 1 | 2 | 3 | 4 | 5 | 6 | 7 | 8 | 9 | 10 |
| Have you perceived a change in the colour of the aligner after cleaning?  (0=no change, 10=remarkable change) | | | | | | | | | |
| 1 | 2 | 3 | 4 | 5 | 6 | 7 | 8 | 9 | 10 |
| How transparent do you consider the aligner to be after cleaning?  (0=not transparent at all, 10=very transparent) | | | | | | | | | |
| 1 | 2 | 3 | 4 | 5 | 6 | 7 | 8 | 9 | 10 |
| How opaque do you consider the aligner to be after cleaning? (0=not opaque at all, 10=very opaque) | | | | | | | | | |
| 1 | 2 | 3 | 4 | 5 | 6 | 7 | 8 | 9 | 10 |
| How do you rate the ease of use of the product? (0=very difficult, 10=very easy) | | | | | | | | | |
| 1 | 2 | 3 | 4 | 5 | 6 | 7 | 8 | 9 | 10 |
| Do you consider the cleaning time to be excessive? (0=not at all, 10=absolutely) | | | | | | | | | |
| 1 | 2 | 3 | 4 | 5 | 6 | 7 | 8 | 9 | 10 |
| How do you rate the cleanliness of the aligner after cleaning? (0=very dirty, 10=very clean) | | | | | | | | | |
| 1 | 2 | 3 | 4 | 5 | 6 | 7 | 8 | 9 | 10 |
| Have you perceived a change in salivation? (0=no change, 10=remarkable change) | | | | | | | | | |
| 1 | 2 | 3 | 4 | 5 | 6 | 7 | 8 | 9 | 10 |
| Do you need to clean the aligner several times a day? (0=not at all, 10=absolutely) | | | | | | | | | |
| 1 | 2 | 3 | 4 | 5 | 6 | 7 | 8 | 9 | 10 |
| Have you perceived a change in the adherence of the aligner after cleaning?  (0=no change, 10=remarkable change) | | | | | | | | | |
| 1 | 2 | 3 | 4 | 5 | 6 | 7 | 8 | 9 | 10 |

**2. Answers**

**Table 1.** Scores related to answers to question 1. *Means with the same letter are not significantly different (p > 0.05).

|  |  | Mean | St Dev | Min | Median | Max | Significance* |
| --- | --- | --- | --- | --- | --- | --- | --- |
| Water | T1 | 3.22 | 3.96 | 0.00 | 1.00 | 10.00 | A |
|  | T2 | 4.56 | 3.36 | 0.00 | 4.00 | 8.00 | A |
|  | T3 | 4.89 | 3.55 | 0.00 | 5.00 | 10.00 | A |
| Soap | T1 | 5.11 | 3.82 | 1.00 | 3.00 | 10.00 | A |
|  | T2 | 6.11 | 2.67 | 3.00 | 6.00 | 10.00 | A |
|  | T3 | 6.67 | 2.96 | 3.00 | 7.00 | 10.00 | A |
| Polident | T1 | 5.22 | 3.73 | 0.00 | 8.00 | 9.00 | A |
|  | T2 | 6.00 | 2.83 | 2.00 | 7.00 | 9.00 | A |
|  | T3 | 6.78 | 2.68 | 3.00 | 8.00 | 10.00 | A |
| Crystal | T1 | 5.22 | 3.42 | 0.00 | 6.00 | 10.00 | A |
|  | T2 | 6.00 | 2.83 | 0.00 | 7.00 | 9.00 | A |
|  | T3 | 6.00 | 2.83 | 0.00 | 7.00 | 9.00 | A |
| Geldis | T1 | 4.33 | 3.64 | 0.00 | 6.00 | 9.00 | A |
|  | T2 | 5.67 | 3.00 | 0.00 | 7.00 | 9.00 | A |
|  | T3 | 5.78 | 2.91 | 0.00 | 7.00 | 9.00 | A |

**Table 2.** Scores related to answers to question 2. *Means with the same letter are not significantly different (p > 0.05).

|  |  | Mean | St Dev | Min | Median | Max | Significance* |
| --- | --- | --- | --- | --- | --- | --- | --- |
| Water | T1 | 7.00 | 2.74 | 1.00 | 8.00 | 10.00 | A |
|  | T2 | 5.44 | 3.05 | 0.00 | 6.00 | 9.00 | A |
|  | T3 | 5.00 | 3.28 | 0.00 | 5.00 | 9.00 | A |
| Soap | T1 | 8.11 | 1.83 | 4.00 | 8.00 | 10.00 | A |
|  | T2 | 7.78 | 2.11 | 3.00 | 8.00 | 10.00 | A |
|  | T3 | 7.44 | 2.70 | 1.00 | 8.00 | 10.00 | A |
| Polident | T1 | 8.56 | 2.55 | 2.00 | 9.00 | 10.00 | A |
|  | T2 | 8.00 | 2.50 | 2.00 | 8.00 | 10.00 | A |
|  | T3 | 7.67 | 2.60 | 2.00 | 8.00 | 10.00 | A |
| Crystal | T1 | 5.22 | 3.23 | 0.00 | 6.00 | 9.00 | A |
|  | T2 | 5.78 | 2.95 | 1.00 | 7.00 | 9.00 | A |
|  | T3 | 5.56 | 2.88 | 1.00 | 6.00 | 9.00 | A |
| Geldis | T1 | 7.67 | 1.32 | 6.00 | 7.00 | 9.00 | A |
|  | T2 | 7.22 | 1.48 | 6.00 | 7.00 | 10.00 | A |
|  | T3 | 7.00 | 1.58 | 5.00 | 7.00 | 10.00 | A |

**Table 3.** Scores related to answers to question 3. *Means with the same letter are not significantly different (p > 0.05).

|  |  | Mean | St Dev | Min | Median | Max | Significance* |
| --- | --- | --- | --- | --- | --- | --- | --- |
| Water | T1 | 4.11 | 4.28 | 0.00 | 2.00 | 10.00 | A |
|  | T2 | 3.89 | 3.79 | 0.00 | 3.00 | 8.00 | A |
|  | T3 | 3.67 | 3.74 | 0.00 | 2.00 | 8.00 | A |
| Soap | T1 | 6.44 | 3.94 | 0.00 | 9.00 | 10.00 | A |
|  | T2 | 6.67 | 3.39 | 0.00 | 8.00 | 10.00 | A |
|  | T3 | 6.78 | 3.42 | 0.00 | 8.00 | 10.00 | A |
| Polident | T1 | 5.89 | 3.55 | 0.00 | 7.00 | 9.00 | A |
|  | T2 | 6.44 | 3.28 | 0.00 | 7.00 | 10.00 | A |
|  | T3 | 6.11 | 3.41 | 0.00 | 7.00 | 10.00 | A |
| Crystal | T1 | 4.67 | 2.96 | 0.00 | 6.00 | 8.00 | A |
|  | T2 | 5.22 | 2.39 | 1.00 | 6.00 | 8.00 | A |
|  | T3 | 5.22 | 2.33 | 1.00 | 6.00 | 8.00 | A |
| Geldis | T1 | 5.22 | 3.42 | 0.00 | 5.00 | 9.00 | A |
|  | T2 | 5.78 | 3.11 | 0.00 | 7.00 | 9.00 | A |
|  | T3 | 5.89 | 3.10 | 0.00 | 7.00 | 9.00 | A |

**Table 4.** Scores related to answers to question 4. *Means with the same letter are not significantly different (p > 0.05).

|  |  | Mean | St Dev | Min | Median | Max | Significance* |
| --- | --- | --- | --- | --- | --- | --- | --- |
| Water | T1 | 1.22 | 2.95 | 0.00 | 0.00 | 9.00 | A |
|  | T2 | 3.22 | 3.23 | 0.00 | 2.00 | 10.00 | A |
|  | T3 | 3.78 | 3.46 | 0.00 | 3.00 | 10.00 | A |
| Soap | T1 | 0.67 | 1.12 | 0.00 | 0.00 | 3.00 | A |
|  | T2 | 1.78 | 1.56 | 0.00 | 2.00 | 5.00 | A |
|  | T3 | 2.33 | 2.24 | 0.00 | 2.00 | 7.00 | A |
| Polident | T1 | 1.22 | 3.31 | 0.00 | 0.00 | 10.00 | A |
|  | T2 | 1.22 | 3.31 | 0.00 | 0.00 | 10.00 | A |
|  | T3 | 1.67 | 3.24 | 0.00 | 0.00 | 10.00 | A |
| Crystal | T1 | 2.78 | 3.27 | 0.00 | 2.00 | 10.00 | A |
|  | T2 | 2.44 | 2.70 | 0.00 | 1.00 | 7.00 | A |
|  | T3 | 2.67 | 2.55 | 0.00 | 2.00 | 7.00 | A |
| Geldis | T1 | 0.44 | 1.01 | 0.00 | 0.00 | 3.00 | A |
|  | T2 | 0.78 | 1.30 | 0.00 | 0.00 | 3.00 | A |
|  | T3 | 0.89 | 1.27 | 0.00 | 0.00 | 3.00 | A |

**Table 5.** Scores related to answers to question 5. *Means with the same letter are not significantly different (p > 0.05).

|  |  | Mean | St Dev | Min | Median | Max | Significance* |
| --- | --- | --- | --- | --- | --- | --- | --- |
| Water | T1 | 2.00 | 3.71 | 0.00 | 0.00 | 9.00 | A |
|  | T2 | 2.56 | 3.47 | 0.00 | 1.00 | 9.00 | A |
|  | T3 | 2.33 | 3.12 | 0.00 | 1.00 | 8.00 | A |
| Soap | T1 | 4.44 | 4.30 | 0.00 | 4.00 | 10.00 | A |
|  | T2 | 4.89 | 3.79 | 0.00 | 4.00 | 10.00 | A |
|  | T3 | 5.00 | 3.87 | 0.00 | 4.00 | 10.00 | A |
| Polident | T1 | 4.22 | 4.41 | 0.00 | 2.00 | 10.00 | A |
|  | T2 | 4.22 | 3.56 | 0.00 | 4.00 | 8.00 | A |
|  | T3 | 3.78 | 3.23 | 0.00 | 3.00 | 8.00 | A |
| Crystal | T1 | 3.89 | 4.11 | 0.00 | 2.00 | 10.00 | A |
|  | T2 | 4.22 | 3.80 | 0.00 | 3.00 | 9.00 | A |
|  | T3 | 4.56 | 3.78 | 0.00 | 4.00 | 9.00 | A |
| Geldis | T1 | 2.56 | 3.47 | 0.00 | 1.00 | 9.00 | A |
|  | T2 | 3.67 | 3.35 | 0.00 | 3.00 | 8.00 | A |
|  | T3 | 3.67 | 3.43 | 0.00 | 3.00 | 8.00 | A |

**Table 6.** Scores related to answers to question 6. *Means with the same letter are not significantly different (p > 0.05).

|  |  | Mean | St Dev | Min | Median | Max | Significance* |
| --- | --- | --- | --- | --- | --- | --- | --- |
| Water | T1 | 6.67 | 2.74 | 1.00 | 7.00 | 10.00 | A |
|  | T2 | 6.56 | 2.70 | 1.00 | 7.00 | 10.00 | A |
|  | T3 | 6.44 | 2.55 | 1.00 | 7.00 | 10.00 | A |
| Soap | T1 | 7.78 | 1.56 | 5.00 | 8.00 | 10.00 | A |
|  | T2 | 7.67 | 1.41 | 5.00 | 8.00 | 10.00 | A |
|  | T3 | 7.67 | 1.41 | 5.00 | 8.00 | 10.00 | A |
| Polident | T1 | 8.33 | 1.58 | 5.00 | 9.00 | 10.00 | A |
|  | T2 | 8.11 | 1.54 | 5.00 | 8.00 | 10.00 | A |
|  | T3 | 7.78 | 1.72 | 5.00 | 8.00 | 10.00 | A |
| Crystal | T1 | 5.89 | 3.14 | 0.00 | 7.00 | 8.00 | A |
|  | T2 | 5.89 | 2.62 | 1.00 | 7.00 | 8.00 | A |
|  | T3 | 6.00 | 2.74 | 1.00 | 7.00 | 9.00 | A |
| Geldis | T1 | 7.22 | 2.28 | 2.00 | 8.00 | 10.00 | A |
|  | T2 | 7.33 | 2.18 | 2.00 | 8.00 | 9.00 | A |
|  | T3 | 7.22 | 2.17 | 2.00 | 8.00 | 9.00 | A |

**Table 7.** Scores related to answers to question 7. *Means with the same letter are not significantly different (p > 0.05).

|  |  | Mean | St Dev | Min | Median | Max | Significance* |
| --- | --- | --- | --- | --- | --- | --- | --- |
| Water | T1 | 2.56 | 3.40 | 0.00 | 1.00 | 8.00 | A |
|  | T2 | 2.89 | 3.22 | 0.00 | 2.00 | 8.00 | A |
|  | T3 | 2.89 | 3.06 | 0.00 | 2.00 | 8.00 | A |
| Soap | T1 | 6.11 | 3.55 | 0.00 | 7.00 | 10.00 | A |
|  | T2 | 6.56 | 2.88 | 0.00 | 7.00 | 10.00 | A |
|  | T3 | 6.56 | 2.88 | 0.00 | 7.00 | 10.00 | A |
| Polident | T1 | 6.33 | 3.74 | 0.00 | 7.00 | 10.00 | A |
|  | T2 | 6.33 | 3.46 | 0.00 | 7.00 | 10.00 | A |
|  | T3 | 6.11 | 3.37 | 0.00 | 7.00 | 10.00 | A |
| Crystal | T1 | 3.44 | 2.79 | 0.00 | 3.00 | 8.00 | A |
|  | T2 | 3.89 | 2.15 | 1.00 | 3.00 | 8.00 | A |
|  | T3 | 3.89 | 2.09 | 1.00 | 4.00 | 8.00 | A |
| Geldis | T1 | 4.56 | 3.54 | 0.00 | 5.00 | 9.00 | A |
|  | T2 | 5.56 | 2.96 | 0.00 | 6.00 | 9.00 | A |
|  | T3 | 5.78 | 2.95 | 0.00 | 6.00 | 9.00 | A |

**Table 8.** Scores related to answers to question 8. *Means with the same letter are not significantly different (p > 0.05).

|  |  | Mean | St Dev | Min | Median | Max | Significance* |
| --- | --- | --- | --- | --- | --- | --- | --- |
| Water | T1 | 1.33 | 2.96 | 0.00 | 0.00 | 9.00 | A |
|  | T2 | 1.89 | 3.22 | 0.00 | 1.00 | 10.00 | A |
|  | T3 | 2.33 | 3.20 | 0.00 | 1.00 | 10.00 | A |
| Soap | T1 | 0.44 | 0.53 | 0.00 | 0.00 | 1.00 | A |
|  | T2 | 1.11 | 1.05 | 0.00 | 1.00 | 3.00 | A |
|  | T3 | 1.22 | 1.20 | 0.00 | 1.00 | 3.00 | A |
| Polident | T1 | 0.11 | 0.33 | 0.00 | 0.00 | 1.00 | A |
|  | T2 | 0.22 | 0.44 | 0.00 | 0.00 | 1.00 | A |
|  | T3 | 0.22 | 0.67 | 0.00 | 0.00 | 2.00 | A |
| Crystal | T1 | 2.56 | 3.32 | 0.00 | 2.00 | 10.00 | A |
|  | T2 | 2.33 | 2.78 | 0.00 | 2.00 | 8.00 | A |
|  | T3 | 2.56 | 2.74 | 0.00 | 2.00 | 8.00 | A |
| Geldis | T1 | 1.00 | 3.00 | 0.00 | 0.00 | 9.00 | A |
|  | T2 | 1.56 | 2.92 | 0.00 | 0.00 | 9.00 | A |
|  | T3 | 1.67 | 2.96 | 0.00 | 0.00 | 9.00 | A |

**Table 9.** Scores related to answers to question 9. *Means with the same letter are not significantly different (p > 0.05).

|  |  | Mean | St Dev | Min | Median | Max | Significance* |
| --- | --- | --- | --- | --- | --- | --- | --- |
| Water | T1 | 2.11 | 3.69 | 0.00 | 0.00 | 9.00 | A |
|  | T2 | 2.25 | 3.62 | 0.00 | 0.00 | 8.00 | A |
|  | T3 | 2.5 | 3.30 | 0.00 | 1.00 | 8.00 | A |
| Soap | T1 | 0.67 | 1.32 | 0.00 | 0.00 | 4.00 | A |
|  | T2 | 1.22 | 1.79 | 0.00 | 0.00 | 5.00 | A |
|  | T3 | 1.56 | 2.07 | 0.00 | 1.00 | 6.00 | A |
| Polident | T1 | 0.00 | 0.00 | 0.00 | 0.00 | 0.00 | A |
|  | T2 | 0.00 | 0.00 | 0.00 | 0.00 | 0.00 | A |
|  | T3 | 0.56 | 1.33 | 0.00 | 0.00 | 4.00 | A |
| Crystal | T1 | 0.56 | 1.01 | 0.00 | 0.00 | 3.00 | A |
|  | T2 | 0.67 | 1.12 | 0.00 | 0.00 | 3.00 | A |
|  | T3 | 1.33 | 2.18 | 0.00 | 0.00 | 6.00 | A |
| Geldis | T1 | 0.22 | 0.44 | 0.00 | 0.00 | 1.00 | A |
|  | T2 | 0.44 | 0.53 | 0.00 | 0.00 | 1.00 | A |
|  | T3 | 0.33 | 0.50 | 0.00 | 0.00 | 1.00 | A |

**Table 10.** Scores related to answers to question 10. *Means with the same letter are not significantly different (p > 0.05).

|  |  | Mean | St Dev | Min | Median | Max | Significance* |
| --- | --- | --- | --- | --- | --- | --- | --- |
| Water | T1 | 4.67 | 3.94 | 0.00 | 4.00 | 10.00 | A |
|  | T2 | 4.89 | 3.82 | 0.00 | 5.00 | 10.00 | A |
|  | T3 | 5.22 | 3.67 | 0.00 | 5.00 | 10.00 | A |
| Soap | T1 | 0.78 | 1.99 | 0.00 | 0.00 | 6.00 | A |
|  | T2 | 1.44 | 2.01 | 0.00 | 1.00 | 6.00 | A |
|  | T3 | 1.78 | 2.05 | 0.00 | 1.00 | 6.00 | A |
| Polident | T1 | 1.33 | 1.87 | 0.00 | 1.00 | 6.00 | A |
|  | T2 | 1.22 | 1.92 | 0.00 | 1.00 | 6.00 | A |
|  | T3 | 1.78 | 1.99 | 0.00 | 1.00 | 6.00 | A |
| Crystal | T1 | 1.56 | 2.55 | 0.00 | 0.00 | 6.00 | A |
|  | T2 | 1.78 | 2.49 | 0.00 | 1.00 | 6.00 | A |
|  | T3 | 2.44 | 2.74 | 0.00 | 1.00 | 6.00 | A |
| Geldis | T1 | 1.56 | 2.74 | 0.00 | 0.00 | 8.00 | A |
|  | T2 | 1.78 | 2.64 | 0.00 | 1.00 | 8.00 | A |
|  | T3 | 1.78 | 2.64 | 0.00 | 1.00 | 8.00 | A |

**Table 11.** Scores related to answers to question 11. *Means with the same letter are not significantly different (p > 0.05).

|  |  | Mean | St Dev | Min | Median | Max | Significance* |
| --- | --- | --- | --- | --- | --- | --- | --- |
| Water | T1 | 1.11 | 2.67 | 0.00 | 0.00 | 8.00 | A |
|  | T2 | 3.33 | 3.57 | 0.00 | 2.00 | 10.00 | A |
|  | T3 | 4.11 | 3.66 | 0.00 | 2.00 | 10.00 | A |
| Soap | T1 | 2.11 | 3.76 | 0.00 | 0.00 | 10.00 | A |
|  | T2 | 3.00 | 3.61 | 0.00 | 2.00 | 10.00 | A |
|  | T3 | 3.22 | 3.73 | 0.00 | 2.00 | 10.00 | A |
| Polident | T1 | 1.22 | 2.39 | 0.00 | 0.00 | 7.00 | A |
|  | T2 | 2.33 | 3.35 | 0.00 | 0.00 | 8.00 | A |
|  | T3 | 2.67 | 3.77 | 0.00 | 0.00 | 8.00 | A |
| Crystal | T1 | 2.56 | 3.36 | 0.00 | 0.00 | 9.00 | A |
|  | T2 | 2.89 | 2.98 | 0.00 | 2.00 | 9.00 | A |
|  | T3 | 3.22 | 3.11 | 0.00 | 3.00 | 9.00 | A |
| Geldis | T1 | 1.13 | 2.10 | 0.00 | 0.00 | 6.00 | A |
|  | T2 | 3.25 | 2.76 | 0.00 | 2.50 | 7.00 | A |
|  | T3 | 3.63 | 3.20 | 0.00 | 3.00 | 9.00 | A |

**Table 12.** Scores related to answers to question 12. *Means with the same letter are not significantly different (p > 0.05).

|  |  | Mean | St Dev | Min | Median | Max | Significance* |
| --- | --- | --- | --- | --- | --- | --- | --- |
| Water | T1 | 8.33 | 2.50 | 2.00 | 9.00 | 10.00 | A |
|  | T2 | 7.22 | 2.28 | 3.00 | 8.00 | 10.00 | A |
|  | T3 | 5.78 | 3.07 | 1.00 | 6.00 | 10.00 | A |
| Soap | T1 | 9.11 | 0.93 | 8.00 | 9.00 | 10.00 | A |
|  | T2 | 8.44 | 1.13 | 7.00 | 8.00 | 10.00 | A |
|  | T3 | 8.11 | 1.27 | 6.00 | 8.00 | 10.00 | A |
| Polident | T1 | 8.33 | 2.06 | 4.00 | 9.00 | 10.00 | A |
|  | T2 | 8.25 | 1.58 | 6.00 | 8.00 | 10.00 | A |
|  | T3 | 8.13 | 1.55 | 6.00 | 8.00 | 10.00 | A |
| Crystal | T1 | 8.56 | 1.24 | 7.00 | 8.00 | 10.00 | A |
|  | T2 | 7.89 | 1.62 | 5.00 | 8.00 | 10.00 | A |
|  | T3 | 7.44 | 1.74 | 5.00 | 7.00 | 10.00 | A |
| Geldis | T1 | 8.75 | 1.04 | 7.00 | 9.00 | 10.00 | A |
|  | T2 | 7.13 | 1.96 | 3.00 | 7.50 | 9.00 | A |
|  | T3 | 6.50 | 2.62 | 1.00 | 7.00 | 9.00 | A |

**Table 13.** Scores related to answers to question 13. *Means with the same letter are not significantly different (p > 0.05).

|  |  | Mean | St Dev | Min | Median | Max | Significance* |
| --- | --- | --- | --- | --- | --- | --- | --- |
| Water | T1 | 2.89 | 3.69 | 0.00 | 1.00 | 9.00 | A |
|  | T2 | 4.25 | 2.76 | 0.00 | 4.50 | 8.00 | A |
|  | T3 | 4.88 | 2.85 | 0.00 | 5.00 | 8.00 | A |
| Soap | T1 | 0.67 | 0.71 | 0.00 | 1.00 | 2.00 | A |
|  | T2 | 1.22 | 0.97 | 0.00 | 2.00 | 2.00 | A |
|  | T3 | 1.56 | 1.24 | 0.00 | 2.00 | 4.00 | A |
| Polident | T1 | 1.11 | 1.45 | 0.00 | 1.00 | 4.00 | A |
|  | T2 | 1.78 | 1.79 | 0.00 | 1.00 | 4.00 | A |
|  | T3 | 1.89 | 1.96 | 0.00 | 1.00 | 5.00 | A |
| Crystal | T1 | 0.89 | 1.45 | 0.00 | 0.00 | 4.00 | A |
|  | T2 | 1.44 | 1.59 | 0.00 | 1.00 | 4.00 | A |
|  | T3 | 2.00 | 1.73 | 0.00 | 3.00 | 4.00 | A |
| Geldis | T1 | 1.75 | 2.66 | 0.00 | 1.00 | 8.00 | A |
|  | T2 | 3.25 | 3.15 | 0.00 | 2.00 | 8.00 | A |
|  | T3 | 3.63 | 3.20 | 1.00 | 2.50 | 9.00 | A |

**Table 14.** Scores related to answers to question 14. *Means with the same letter are not significantly different (p > 0.05).

|  |  | Mean | St Dev | Min | Median | Max | Significance* |
| --- | --- | --- | --- | --- | --- | --- | --- |
| Water | T1 | 9.44 | 0.73 | 8.00 | 10.00 | 10.00 | A |
|  | T2 | 9.00 | 1.00 | 8.00 | 9.00 | 10.00 | A |
|  | T3 | 9.00 | 1.00 | 8.00 | 9.00 | 10.00 | A |
| Soap | T1 | 8.78 | 1.64 | 5.00 | 9.00 | 10.00 | A |
|  | T2 | 8.78 | 1.64 | 5.00 | 9.00 | 10.00 | A |
|  | T3 | 8.78 | 1.64 | 5.00 | 9.00 | 10.00 | A |
| Polident | T1 | 8.89 | 1.69 | 5.00 | 10.00 | 10.00 | A |
|  | T2 | 9.11 | 1.36 | 6.00 | 10.00 | 10.00 | A |
|  | T3 | 9.00 | 1.50 | 6.00 | 10.00 | 10.00 | A |
| Crystal | T1 | 8.67 | 1.12 | 7.00 | 8.00 | 10.00 | A |
|  | T2 | 8.78 | 1.09 | 7.00 | 9.00 | 10.00 | A |
|  | T3 | 8.78 | 1.09 | 7.00 | 9.00 | 10.00 | A |
| Geldis | T1 | 8.50 | 2.07 | 4.00 | 9.00 | 10.00 | A |
|  | T2 | 8.25 | 2.19 | 4.00 | 9.00 | 10.00 | A |
|  | T3 | 8.13 | 2.23 | 4.00 | 9.00 | 10.00 | A |

**Table 15.** Scores related to answers to question 15. *Means with the same letter are not significantly different (p > 0.05).

|  |  | Mean | St Dev | Min | Median | Max | Significance* |
| --- | --- | --- | --- | --- | --- | --- | --- |
| Water | T1 | 1.67 | 2.65 | 0.00 | 0.00 | 8.00 | A |
|  | T2 | 1.56 | 2.24 | 0.00 | 1.00 | 7.00 | A |
|  | T3 | 1.56 | 2.24 | 0.00 | 1.00 | 7.00 | A |
| Soap | T1 | 1.22 | 1.48 | 0.00 | 0.00 | 3.00 | A |
|  | T2 | 1.33 | 1.41 | 0.00 | 1.00 | 3.00 | A |
|  | T3 | 1.33 | 1.41 | 0.00 | 1.00 | 3.00 | A |
| Polident | T1 | 3.44 | 3.32 | 0.00 | 3.00 | 10.00 | A |
|  | T2 | 3.44 | 3.32 | 0.00 | 3.00 | 10.00 | A |
|  | T3 | 3.44 | 3.32 | 0.00 | 3.00 | 10.00 | A |
| Crystal | T1 | 4.67 | 3.50 | 0.00 | 6.00 | 10.00 | A |
|  | T2 | 4.78 | 3.56 | 0.00 | 6.00 | 10.00 | A |
|  | T3 | 4.89 | 3.69 | 0.00 | 6.00 | 10.00 | A |
| Geldis | T1 | 1.63 | 3.42 | 0.00 | 0.50 | 10.00 | A |
|  | T2 | 1.63 | 3.42 | 0.00 | 0.50 | 10.00 | A |
|  | T3 | 1.63 | 3.42 | 0.00 | 0.50 | 10.00 | A |

**Table 16.** Scores related to answers to question 16. *Means with the same letter are not significantly different (p > 0.05).

|  |  | Mean | St Dev | Min | Median | Max | Significance* |
| --- | --- | --- | --- | --- | --- | --- | --- |
| Water | T1 | 6.89 | 3.06 | 0.00 | 8.00 | 10.00 | A |
|  | T2 | 5.78 | 3.53 | 0.00 | 7.00 | 10.00 | A |
|  | T3 | 5.33 | 3.46 | 0.00 | 7.00 | 10.00 | A |
| Soap | T1 | 8.78 | 0.83 | 8.00 | 9.00 | 10.00 | A |
|  | T2 | 8.11 | 0.78 | 7.00 | 8.00 | 9.00 | A |
|  | T3 | 7.89 | 0.78 | 7.00 | 8.00 | 9.00 | A |
| Polident | T1 | 8.89 | 1.27 | 7.00 | 9.00 | 10.00 | A |
|  | T2 | 8.78 | 0.97 | 8.00 | 8.00 | 10.00 | A |
|  | T3 | 8.67 | 1.12 | 7.00 | 8.00 | 10.00 | A |
| Crystal | T1 | 7.89 | 1.45 | 6.00 | 8.00 | 10.00 | A |
|  | T2 | 7.78 | 1.64 | 5.00 | 8.00 | 10.00 | A |
|  | T3 | 7.44 | 1.51 | 5.00 | 8.00 | 9.00 | A |
| Geldis | T1 | 8.13 | 1.46 | 6.00 | 8.00 | 10.00 | A |
|  | T2 | 7.75 | 1.28 | 6.00 | 7.50 | 10.00 | A |
|  | T3 | 7.50 | 0.93 | 6.00 | 7.50 | 9.00 | A |

**Table 17.** Scores related to answers to question 17. *Means with the same letter are not significantly different (p > 0.05).

|  |  | Mean | St Dev | Min | Median | Max | Significance* |
| --- | --- | --- | --- | --- | --- | --- | --- |
| Water | T1 | 1.78 | 2.59 | 0.00 | 0.00 | 7.00 | A |
|  | T2 | 1.44 | 2.07 | 0.00 | 0.00 | 6.00 | A |
|  | T3 | 1.78 | 2.05 | 0.00 | 2.00 | 6.00 | A |
| Soap | T1 | 1.78 | 2.99 | 0.00 | 0.00 | 7.00 | A |
|  | T2 | 1.56 | 2.83 | 0.00 | 0.00 | 7.00 | A |
|  | T3 | 1.67 | 2.83 | 0.00 | 0.00 | 7.00 | A |
| Polident | T1 | 0.67 | 1.32 | 0.00 | 0.00 | 3.00 | A |
|  | T2 | 0.56 | 0.88 | 0.00 | 0.00 | 2.00 | A |
|  | T3 | 0.56 | 0.88 | 0.00 | 0.00 | 2.00 | A |
| Crystal | T1 | 1.33 | 2.65 | 0.00 | 0.00 | 8.00 | A |
|  | T2 | 1.44 | 2.70 | 0.00 | 0.00 | 8.00 | A |
|  | T3 | 1.44 | 2.70 | 0.00 | 0.00 | 8.00 | A |
| Geldis | T1 | 2.56 | 3.43 | 0.00 | 0.00 | 8.00 | A |
|  | T2 | 2.56 | 3.43 | 0.00 | 0.00 | 8.00 | A |
|  | T3 | 2.56 | 3.43 | 0.00 | 0.00 | 8.00 | A |

**Table 18.** Scores related to answers to question 18. *Means with the same letter are not significantly different (p > 0.05).

|  |  | Mean | St Dev | Min | Median | Max | Significance* |
| --- | --- | --- | --- | --- | --- | --- | --- |
| Water | T1 | 4.56 | 3.84 | 0.00 | 6.00 | 10.00 | A |
|  | T2 | 5.89 | 3.33 | 0.00 | 7.00 | 10.00 | A |
|  | T3 | 6.22 | 3.53 | 0.00 | 7.00 | 10.00 | A |
| Soap | T1 | 2.78 | 3.11 | 0.00 | 2.00 | 8.00 | A |
|  | T2 | 3.33 | 3.20 | 0.00 | 4.00 | 8.00 | A |
|  | T3 | 3.78 | 3.27 | 0.00 | 4.00 | 8.00 | A |
| Polident | T1 | 2.11 | 2.71 | 0.00 | 1.00 | 7.00 | A |
|  | T2 | 2.22 | 2.59 | 0.00 | 2.00 | 7.00 | A |
|  | T3 | 2.33 | 2.60 | 0.00 | 2.00 | 7.00 | A |
| Crystal | T1 | 2.56 | 3.05 | 0.00 | 2.00 | 8.00 | A |
|  | T2 | 2.78 | 3.07 | 0.00 | 2.00 | 8.00 | A |
|  | T3 | 2.67 | 2.96 | 0.00 | 2.00 | 8.00 | A |
| Geldis | T1 | 2.78 | 3.49 | 0.00 | 2.00 | 10.00 | A |
|  | T2 | 3.67 | 3.20 | 0.00 | 4.00 | 10.00 | A |
|  | T3 | 4.00 | 3.32 | 0.00 | 4.00 | 10.00 | A |

**Table 19.** Scores related to answers to question 19. *Means with the same letter are not significantly different (p > 0.05).

|  |  | Mean | St Dev | Min | Median | Max | Significance* |
| --- | --- | --- | --- | --- | --- | --- | --- |
| Water | T1 | 1.56 | 2.65 | 0.00 | 0.00 | 7.00 | A |
|  | T2 | 1.67 | 2.92 | 0.00 | 0.00 | 8.00 | A |
|  | T3 | 1.67 | 2.92 | 0.00 | 0.00 | 8.00 | A |
| Soap | T1 | 0.89 | 2.32 | 0.00 | 0.00 | 7.00 | A |
|  | T2 | 0.89 | 2.03 | 0.00 | 0.00 | 6.00 | A |
|  | T3 | 0.78 | 1.72 | 0.00 | 0.00 | 5.00 | A |
| Polident | T1 | 0.44 | 1.01 | 0.00 | 0.00 | 3.00 | A |
|  | T2 | 0.56 | 1.33 | 0.00 | 0.00 | 4.00 | A |
|  | T3 | 0.56 | 1.33 | 0.00 | 0.00 | 4.00 | A |
| Crystal | T1 | 1.00 | 2.00 | 0.00 | 0.00 | 5.00 | A |
|  | T2 | 1.22 | 1.86 | 0.00 | 0.00 | 4.00 | A |
|  | T3 | 1.22 | 1.72 | 0.00 | 0.00 | 4.00 | A |
| Geldis | T1 | 0.22 | 0.44 | 0.00 | 0.00 | 1.00 | A |
|  | T2 | 0.56 | 1.33 | 0.00 | 0.00 | 4.00 | A |
|  | T3 | 0.56 | 1.33 | 0.00 | 0.00 | 4.00 | A |
